# An Improved *in vitro* Model of Cortical Tissue

**DOI:** 10.3389/fnins.2019.01349

**Published:** 2019-12-17

**Authors:** Aaron Gilmour, Laura Poole-Warren, Rylie A. Green

**Affiliations:** ^1^Graduate School of Biomedical Engineering, University of New South Wales, Sydney, NSW, Australia; ^2^Clem Jones Centre for Neurobiology and Stem Cell Research, Menzies Health Institute Queensland, Griffiths University, Gold Coast, QLD, Australia; ^3^Department of Bioengineering, Imperial College London, London, United Kingdom

**Keywords:** brain machine interface, *in vitro* prediction, CNS, cell culture, neural interface response

## Abstract

Intracortical electrodes for brain–machine interfaces rely on intimate contact with tissues for recording signals and stimulating neurons. However, the long-term viability of intracortical electrodes *in vivo* is poor, with a major contributing factor being the development of a glial scar. *In vivo* approaches for evaluating responses to intracortical devices are resource intensive and complex, making statistically significant, high throughput data difficult to obtain. *In vitro* models provide an alternative to *in vivo* studies; however, existing approaches have limitations which restrict the translation of the cellular reactions to the implant scenario. Notably, there is no current robust model that includes astrocytes, microglia, oligodendrocytes and neurons, the four principle cell types, critical to the health, function and wound responses of the central nervous system (CNS). In previous research a co-culture of primary mouse mature mixed glial cells and immature neural precursor cells were shown to mimic several key properties of the CNS response to implanted electrode materials. However, the method was not robust and took up to 63 days, significantly affecting reproducibility and widespread use for assessing brain-material interactions. In the current research a new co-culture approach has been developed and evaluated using immunocytochemistry and quantitative polymerase chain reaction (qPCR). The resulting method reduced the time in culture significantly and the culture model was shown to have a genetic signature similar to that of healthy adult mouse brain. This new robust CNS culture model has the potential to significantly improve the capacity to translate *in vitro* data to the *in vivo* responses.

## Introduction

Investigating the biocompatibility of brain interfacing devices using animal models is expensive, time consuming (Gilmour et al., 2016) and data yield from each animal can be limited by the tissue processing and histological methods used within a study (Woolley et al., 2011). However, existing *in vitro* models for investigating central nervous system (CNS)-device interactions are not a viable alternative, as they poorly represent the complex cell interactions within the CNS and provide little information on the expected *in vivo* response (Horvath et al., 2016; Belle et al., 2018). Despite this, cell culture is a powerful technique for high-throughput studies, enabling parallel assessment across a large number of variables (Astashkina et al., 2012; Zang et al., 2012). An ideal solution is a cell culture model with enough complexity to enable useful insight into implant performance, while not compromising on capacity to trial multiple variables.

For neural cell culture models to be mimetic of the CNS in health and disease, mimicking cell–cell interactions is essential. Interactions both within and between individual glial and neural cell types are critical for the development, function and dysfunction of the CNS (Jäkel and Dimou, 2017). The astrocyte–microglia interaction is the most notable cell–cell interaction and it is pivotal in development, normal function, and response to damage (Liddelow et al., 2017; Yates, 2017). Astrocytes and microglia perform multiple roles in CNS development, ongoing health, and degenerative disease (Burda and Sofroniew, 2014; Pekny and Pekna, 2014; Ferreira and Bernardino, 2015; Sofroniew, 2015; Ziebell et al., 2015; Burda et al., 2016; Liddelow and Barres, 2017). Importantly, the functions of these cells evolve during development undergoing dynamic genotypic and phenotypic changes which are integral to the development of the CNS (see Reemst et al., 2016; Hasel et al., 2017 for in depth reviews). Glial cells change roles from promoting development of neural networks and myelination, to maintaining the complex function of the adult CNS. In response to injury in the mature CNS, glial cells within the wound parenchyma transition to a reactive state (Silver and Miller, 2004; Anderson et al., 2014; Gilmour et al., 2016). In this reactive state mature glial cells produce an environment which does not support redevelopment of neural networks, inhibiting neuronal cell migration and axonal growth (Smith et al., 1990; Canning et al., 1996; Fawcett and Asher, 1999; Faulkner, 2004; Sofroniew, 2009; Cregg et al., 2014; Burda et al., 2016). In contrast, immature glial cells from fetal or neonatal origins lack the ability to undergo reactive gliosis-like reactions *in vivo* and *in vitro* (Schwartz et al., 1989; Wu and Schwartz, 1998).

A number of mixed glial and neuronal cultures have been developed in an attempt to incorporate complex cell behaviors into *in vitro* models (Potter and DeMarse, 2001; Polikov et al., 2006; Thomson et al., 2008; Nash et al., 2011b; Boomkamp et al., 2012; Sommakia et al., 2014). It is expected that this complexity introduces improved alignment with the *in vivo* CNS cell response. However, these culture models often have intricate, multistep methodologies (Polikov et al., 2009), are extremely sensitive to minor modifications and require additional stimulating factors to induce reactive gliosis, limiting their value as a high-throughput assessment tool (Gilmour et al., 2016). Current models have a second limitation whereby the apparent upregulation of glial fibrillary acidic protein (GFAP) and Iba1 in astrocytes and microglia respectively in response to insult does not impact on neural health and regrowth (Polikov et al., 2006; Sommakia et al., 2014). The maturity of glial cells and their relative ability to undergo reactive gliosis has implications for the development and use of complex culture models for modeling CNS and effects of injury. In brain injury and device interactions, scar tissue is formed with glial cells being the dominant component. These cells modulate neuron and oligodendrocyte function, survival, or dieback in the surrounding tissues (Sofroniew, 2009; Burda and Sofroniew, 2014; Burda et al., 2016). In rodents, astrocytes start to express mature genotypes and phenotypes after 3–4 weeks postnatal development (Yang et al., 2013a; Reemst et al., 2016; Hasel et al., 2017) which aligns with the end of the major period of astrogenesis. In contrast the relative maturity of the glial cell populations in prior cultures (Polikov et al., 2006; Sommakia et al., 2014) is equivalent to postnatal days 7–14 (Reemst et al., 2016), at which age rodents are still undergoing neurological development. To achieve adequate glia maturity in these cultures it is estimated that glia would need to be cultured for at least 35 days. It was therefore hypothesized that a more mature population of glial cells are required to enable a CNS culture model with capacity to respond appropriately to injury and implants. The objective of this research was to develop a simple, robust and validated model of the mature rodent CNS. Such a culture could be used for better understanding cell–cell interactions in the CNS, and for mechanistic investigations into CNS injury, repair, and interactions with neural devices.

Co-culture models have been developed to enable understanding and probing of specific glial–neural or glial–glial cell interactions (Banker and Cowan, 1977; Ishikawa et al., 1996; Plenz and Aertsen, 1996; Nakanishi et al., 1999; Flanagan et al., 2002; Faria et al., 2006; Cullen et al., 2007; Wanner et al., 2008; Shimizu et al., 2011; Bogdanowicz and Lu, 2013; van Duinen et al., 2015) of defined cell populations. Previous research (Gilmour, 2018) identified mixed glial cells (MGCs) derived from neonatal mice and cultured for 21 days prior to co-culture generated a glial cell population which was capable of reactive gliosis. Co-culture can be approached by either combining cells in a single concurrent plating step or by staggering the plating to enable one population to develop, prior to addition of the second population. Previous attempts to combine glia and neurons have generally focused on step-wise combinations. One such approach has been the continuous culture of glial cells until they obtain maturity, followed by direct co-culture of neural progenitors (Gilmour, 2018). Despite showing that this culture method develops neural networks which respond to injury at the glial and neuronal level, there are a number of shortcomings limiting this method. First to obtain mature neural networks a continuous culture timeline of ≥ 45 days was required. Second, reproducibility which included failure to obtain time mated embryos at the correct time point (≈66% of failures), poor growth of MGCs after passage (≈15%) and less commonly contaminating cells overgrowing MGC cultures after passaging (≈10%) with an overall failure rate of ≈86%. As such, a more flexible and time efficient method is required to enable complex co-culture of MGCs in combination with neuroprogenitor cells. To address the long culture times and potential for mismatch in time mating, this study proposed the use of frozen mature glial populations that can be stored and reanimated to ensure flexibility and minimization of culture timeframes.

## Materials and Methods

All the chemicals and biological materials were obtained from Sigma-Aldrich (Australia) unless otherwise stated. MCG media consisted of 10% fetal calf serum, in DMEM with L-glutamine. DMMC and co-cultures used three types of media previously described in Thomson et al. (2008), being plating media (PM), defined media with insulin (DfM + I) and defined media without insulin (DfM).

### Co-culture Methodologies

Co-cultures were formed through the combination of 30% MGC and 70% DMMC cells. Once in co-culture format they were fed three times per week with DfM + I for the first 12 days then transitioned to DfM thereafter. Co-cultures were grown on PLL coated glass for developing and assessing the baseline performance of the methods relative to both whole brain extract (for qPCR) and the DMMC cultures as developed by Sorensen et al. (2008) and Thomson et al. (2008).

### Primary Mixed Glia Culture (MGC)

All animal procedures were conducted in accordance with University of New South Wales animal ethics protocols (ACEC 13/44A). Postnatal 1–3 day old mouse pups were euthanized by exposure to excess gaseous isoflurane followed by decapitation. The isolation and culture of MGCs was performed as previously published in Goding et al. (2015), with the following modifications. Cultures were maintained until 80% confluence (approximately 7–10 days) in poly-L-lysine (134 ug mL^–1^) coated T75 tissue culture flasks. Once confluent cultures were trypsinised then frozen in DMEM + 10% FBS with the addition of 10% DMSO. Briefly, cultures were rinsed twice with PBS (without cations) then incubated with 3 mL 0.25% trypsin for 5 min. Trypsin was deactivated by the addition of DMEM + 10% FBS. The resulting cell suspension was centrifuged for 5 min at 290 *g*. Cell concentration was determined with a hemocytometer and diluted with DMEM + 10% FBS to achieve 2^∗^10^^6^ cells mL^–1^ in freezing media.

### Dissociated Mixed Myelinating Culture (DMMC)

Dissociated mixed myelinating culture (DMMCs) were produced using the methods developed in Thomson et al. (2008) with minor modifications. Briefly, gestational day 13.5 pregnant mice were euthanized by an overdose of isoflurane followed by cervical dislocation. Embryos were extracted, spinal cords were removed and stripped of meninges. Harvested spinal cords were dissociated manually, followed by 20 min in 0.25% trypsin EDTA with 0.1% w/v type 1 collagenase. Stop digestion mix was added [40 μg mL^–1^ DNase, 250 μg mL^–1^ trypsin inhibitor, 3 mg mL^–1^ bovine serum albumin fraction V (BSA-V) dissolved in Leibovitz’s L15 (Thermo Fisher Scientific, Australia)]. The cell suspension was then passed three times though a 21G needle followed by two times through a 23G needle. The cell suspension was diluted in PM and centrifuged at 290 *g* for 5 min. The cell pellet was resuspended in PM and cell counting was conducted with a hemocytometer. The DMMC cell suspension was then plated out at 1.5^∗^10^6 cells cm^–1^ onto PLL coated coverslips. After 2 h the culture media was topped up to 500 μL PM with 500 μL DfM + I. Alternatively the cell suspension was used in co-cultures as described below. Cultures were fed three times per week by replacing 50% of the media, using DfM + I for the first 12 days followed by DfM thereafter.

### Layered Co-culture

Time mating was undertaken to obtain E13.5 embryos for tissue harvesting for DMMC cultures. On the day a successful plug was noted frozen MGC were thawed in a 37°C water bath, once thawed the cell suspension was diluted with DMEM + 10% FBS then centrifuged at 290 *g* for 3 min. Revived cells were placed in a PLL coated T75 flask and cultured for 2 days in DMEM + 20% FBS then changed to 10% FBS. After 4 days MGC cultures were passaged and plated at 4^∗^10^^4^ cells cm^–2^ in 1 mL of DMEM + 10% FBS onto PLL coated glass coverslips. Once DMMC were harvested, all media was removed from MGC cultures and DMMC were plated on top in 500 μL of PM at 1.1^∗^10^^5^ cells cm^–1^. Cultures were then fed as per DMMC protocol above.

### Concurrent Co-culture

Mice were time mated and MGC cultures were revived as above, however MGC were not thawed until day 9.5 of pregnancy (4 days prior to embryonic spinal cord harvest). After spinal cord tissue was harvested, dissociated and suspended at 4.4^∗^10^^5^ cells mL^–1^ in plating media, MGC cultures were passaged from the T75 flasks and resuspended to a concentration of 1.6^∗^10^^5^ cells mL^–1^. The MGC and DMMC cell suspensions were mixed 1:1 resulting in a final concentration of 3^∗^10^^5^ cells mL^–1^, 500 μL of cell suspension was plated per coverslip. Cultures were maintained as described for DMMC above.

### Immunocytochemistry and Image Analysis

Cultures were fixed at 21, 28, and 35 days in 4% w/v formaldehyde and processed for microscopy. All primary and secondary antibodies were diluted in blocking buffer, immediately prior to use. Secondary antibodies were raised in goat and conjugated to either Dylight^®^ or Alexa Fluor^®^ 405, 488, 555, and 647 nm fluorophores diluted at 1:200. Primary antibodies were against GFAP (Abcam; ab134436), Iba1 (Wako; 019-19741, RRID: AB_839504) 200 kDa heavy chain neurofilament (Abcam; ab7795, RRID: AB_306084) (H-NF) and proteolipid protein (PLP/DM20) from a hybridoma (RRID: AB_2341144) (Jung et al., 1996).

All images were acquired using a Zeiss 780 laser scanning microscope (LSM) with a Plan-Apochromat 20x/0.8 M27 objective. Non-overlapping regions were captured as z-stacks, with 5 areas per sample. Images were post-processed with ImageJ software (ImageJ 1.50e, National Institutes of Health, United States) implemented on Java 1.8.0_11 (64-bit). Individual channels were deconvolved with 15 iterations of the Richardson-Lucy algorithm implemented via “DevonvolutionLab” plugin (Soltys et al., 2001) with a theoretical point spread function (PSF) and minimal intensity background subtraction. A theoretical PSF was generated with the “Diffraction PSF 3D” plugin for ImageJ to match the dimensions of the acquired images.

The N-NF and PLP/DM20 channels were processed for colocalization to assess the level of interaction under different culture conditions. Colocalization was performed the with Coloc 2 plugin with the default settings. Threshold values generated from Coloc 2 were used as thresholds for the binary conversion of Z-stacks. Z-stacks were converted into maximum intensity projections and total coverage of each channel was expressed as a fraction of the total area in μm^2^.

### Statistical Analysis

Statistical analysis was performed in GraphPad Prism 7.03 (GraphPad Software, La Jolla, CA, United States), all data sets were tested for outliers using ROUT method *Q* = 0.1 (99% confidence that data point is an outlier). A one-way ANOVA followed by Tukey’s multiple comparisons test with a *p* < 0.05 were considered as significant.

### Quantitative PCR

Cultures for messenger ribonucleic acid (mRNA) extraction were rinsed 1x with ice cold DPBS and processed with the ReliaPrep^TM^ RNA cell miniprep system (Promega, Australia) following manufactures instructions. The final RNA extract was eluted into 30 μL of RNase free water. RNA was stored frozen at −80°C until conversion. 5 μL of RNA from each experimental triplicate was pooled, then 10 μL of the pooled RNA for each condition was converted into first strand complementary deoxyribonucleic acid (cDNA) using a High-Capacity cDNA Reverse Transcription Kit (Applied Biosystems, Thermo Fisher, Australia) following manufactures instructions using Bio-Rad C1000 thermal cycler (Bio Rad, Australia). The resulting cDNA was diluted to a total final volume of 100 μL and stored at −80°C prior to qPCR. Primers pairs (see Table 1) were designed with the assistance of Primer-Blast software (Ye et al., 2012) all primer sequences were then cross checked with Beacon Designer Free online tool to identify possible dimers and hairpins.

**TABLE 1:** Primer pairs for qPCR.

**Gene**	**Description**	**Primer sequence (5′ to 3′)**	**Product length**
GAPDH	Reference gene 1	F**′** – AGTGGCAAAGTGGAGATT	83
		R**′** – GTGGAGTCATACTGGAACA	
18s	Reference gene 2	F**′** – TGAGAAGTTCCAGCACATT	75
		R**′** – GTGATGGCGAAGGCTATT	
Iba1	Ionized calcium-binding adapter 1	F**′** – ATACAGCAATGATGAGGAT	111
		R**′** – ATTCGCTTCAAGGACATA	
GFAP	Glial fibrillary acidic protein	F**′** – TCATCCTTGTTGTTATGG	79
		R**′** – CTGTCTGAATTGTTGTCT	
Cdk5	Cyclin dependent kinase 5	F**′** – TCTTCCGACTGCTAGGGACA	219
		R**′** – CAGAGAAGTAGGGGTGCTGC	
Cdk5r1	Cyclin dependent kinase 5 regulatory subunit 1	F**′** – CATAGTTCAGGATTGGATT	174
		R**′** – TTAGCAGTATCGGATGTA	
PLP	Proteolipid protein	F**′** – TCTTCTTGCCATCAGTAG	128
		R**′** – ATGCTATATTGCTCTGCTA	

Quantitative PCR (qPCR) was run on a CFX384 Touch^TM^ real-time PCR detection System (Bio-Rad, Australia) and output was analyzed with Bio-Rad CFX Mastro Software package (Bio-Rad). Power SYBR^TM^ Green PCR master mix (Applied Biosystems, Thermo Fisher, Australia) was used, following manufacturer instructions, reaction volume was set to 10 μL with 2 μL of template used per well with final primer concentrations of 500 nM. Reaction cycles were repeated 40 times followed by melt curve as in Table 2. Each template was run in triplicate for all genes assessed. Relative fold change calculations and statistics were calculated with two reference genes using inbuilt analysis software package.

**TABLE 2:** qPCR reaction cycle settings.

Enzyme activation	95°C	10 min	
Cycle parameters	95°C	15 s	Denature
Repeat 40x	48°C	30 s	Anneal
	70°C	30 s	Extend
Melt curve	70°C–95°C	Read every 0.5°C	

## Results

### Morphological Properties and Interactions

**FIGURE 1:**
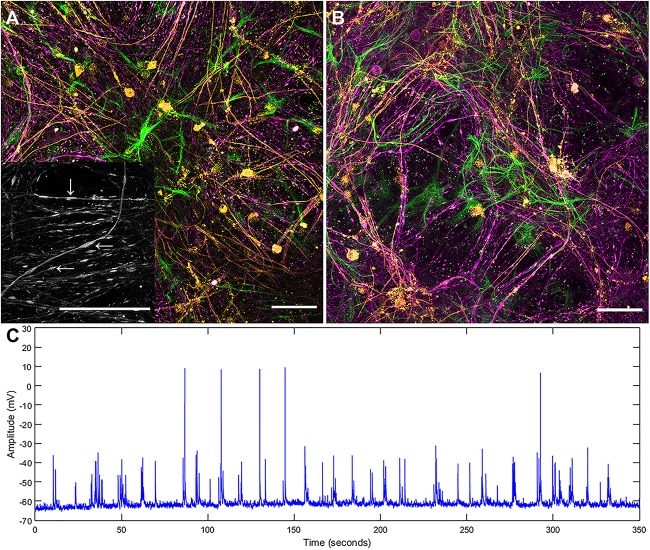
Maximum intensity projections of successful continuous co-culture grown of **(A)** glass and **(B)** platinum surfaces. Co-cultures were grown for 21 days, displaying myelinated neural networks with an abundance of astrocytes. Inset in **(A)** is a 2x enlargement of the underlying H-NF channel only. Although the axons show accumulations of H-NF (swellings along the axons indicated by arrows in inset) which suggests neurodegeneration is occurring (Dale and Garcia, 2012). **(C)** Representative patch clamp recording of spontaneous activity in a control culture. Green – GFAP, Orange – PLP/DM20 and Magenta – H-NF (Scale bar = 50 μm).

Both the layered and concurrent co-culture methods resulted in dense myelinated neural networks which grew for 35 days (a targeted approach to ensure that the original predicted 21 day time period was sufficient for neural network maturation). Both co-culture methods resulted in significantly increased reliability and repeatability over the original continuous co-culture method, shown in Figure 1. The continuous co-culture had an 86% failure rate compared to 100% success rate of both the concurrent and layered methods investigated here. Additionally, accumulations of H-NF were noted in the continuous co-cultures which indicated neural degeneration (see inset Figure 1A), this was not observed in either the layered or concurrent cultures at 35 days.

**FIGURE 2:**
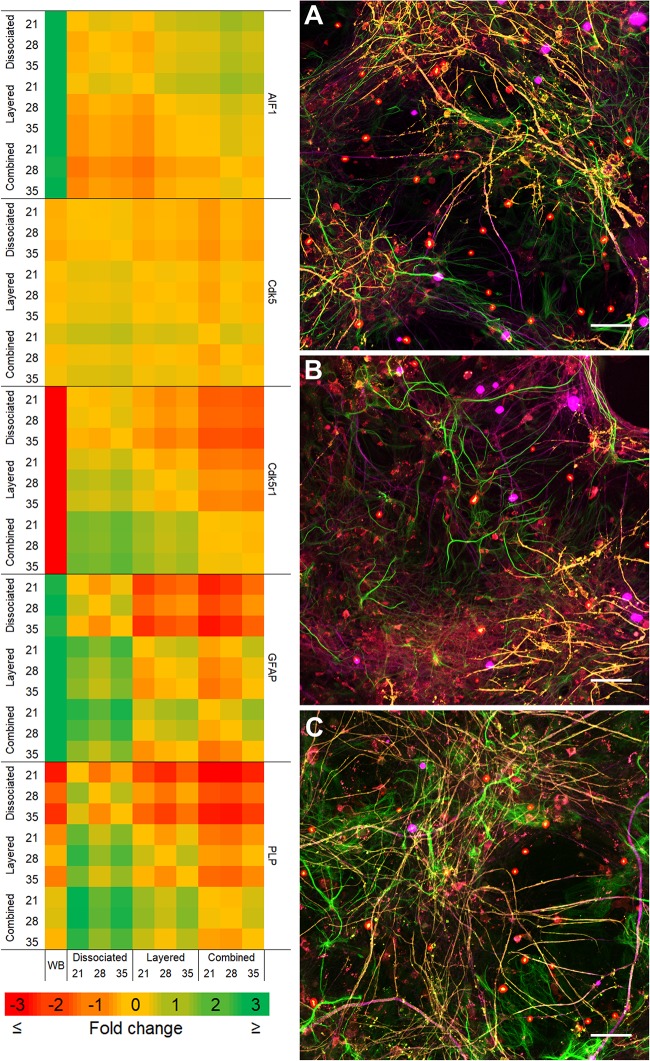
Heat map of qPCR gene expression comparisons between all culture types and whole brain (WB) extract. Maximum intensity projections of composite images of DMMC **(A)** and co-cultures layered **(B)** and concurrent **(C)** at 35 days. Red – Iba1, Green – GFAP, Orange – PLP/DM20 and Magenta – H-NF (*n* = 3, Scale bar = 50 μm). Large saturated round structures are artifacts and were excluded from analysis.

**FIGURE 3:**
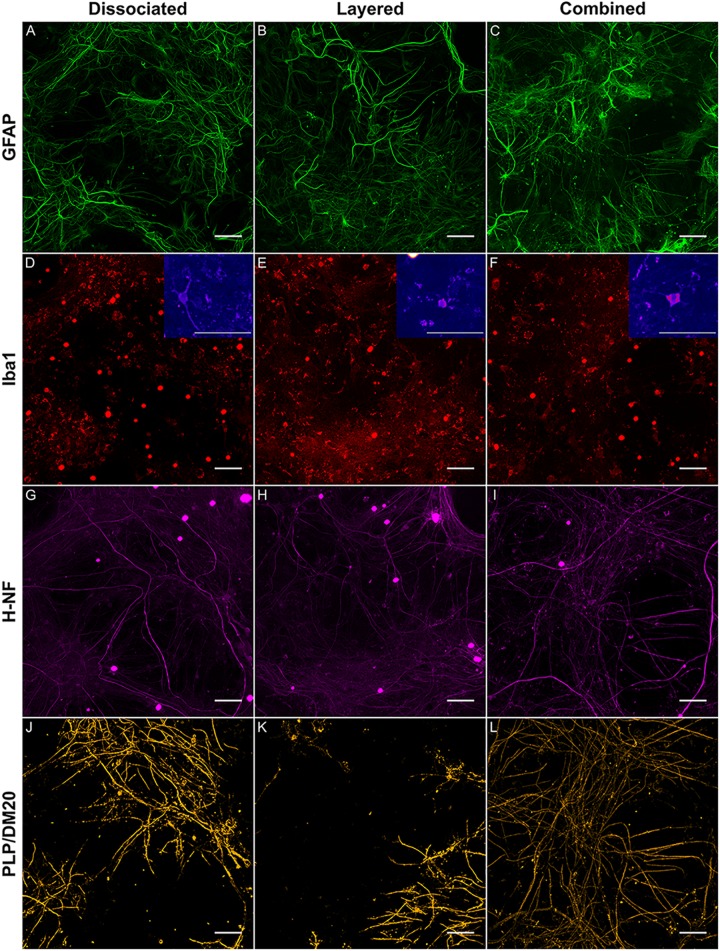
Representative maximum intensity projections comparing the layered and concurrent co-cultures to the DMMC method. GFAP **(A–C)** staining in astrocytes, Iba1 **(D–F)** staining in microglia, H-NF **(G–H)** staining in axons and PLP/DM20 **(J–L)** staining mature myelin, Insets in **D–F** show enlargements of the ramified microglia morphologies present in the cultures. Large saturated round structures Iba1 and H-NF **(D–I)** panels are artefacts and were excluded from analysis.

Comparison of the two co-culture methods with the DMMC culture at the phenotypic and genotypic levels indicate that the concurrent co-culture method resulted in greater numbers of myelinated neural axonal processes and these cultures also expressed significantly higher levels of genes associated with phosphorylated neurofilament and myelin production. Figure 2 shows an overview heat map comparing fold difference in gene expression at each time point and condition. Representative composite images of DMMC and co-cultures at 35 days are shown in Figures 3A–C.

The complexity imparted to the DMMC and co-cultures by including all the major cell types of the CNS resulted in a morphologically diverse and intertwined distribution of GFAP positive astrocytes. Figures 3A–C shows the GFAP morphology in the astrocytes at 35 days in culture. The morphologies present in each culture type were variable across each sample, with fibrous, stellate, and protoplasmic being the most dominant morphologies. When assessed in isolation the GFAP morphologies appeared random, however the three dominant morphologies occupied different domains when the astrocyte proximity with the other cell types were taken into consideration. Protoplasmic astrocytes were predominantly found at the interface of the culture and growth surface. Stellate astrocytes were associated with multiple nerve fibers, and the fibrous astrocytes were aligned with bundles of parallel axons. These cell-cell related morphologies are representative of *in vivo* interactions previously described for the different cell types (Oberheim et al., 2006, 2012; Wang and Bordey, 2008; Sofroniew and Vinters, 2010).

**FIGURE 4:**
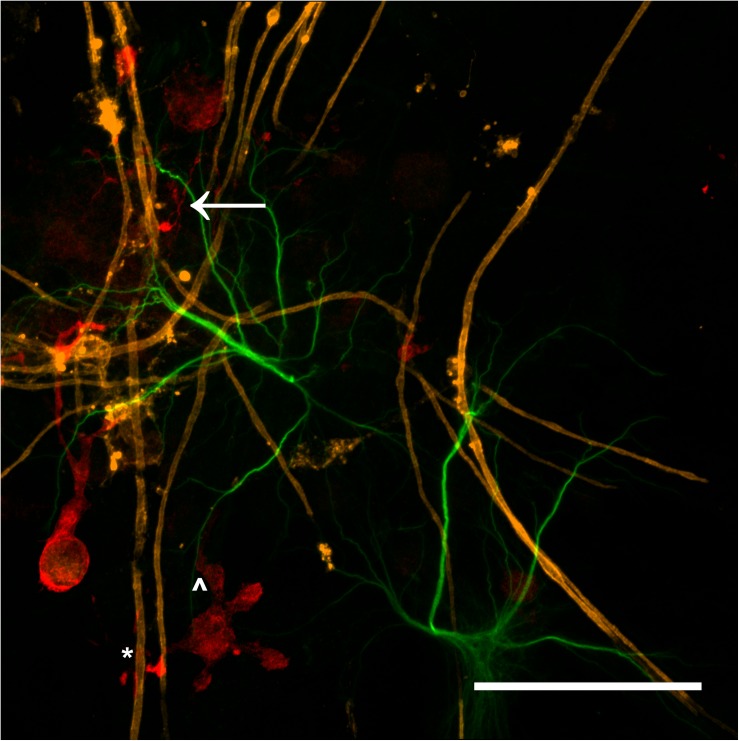
Maximum intensity projection of a 100x magnification tile scan from a concurrent co-culture demonstrating the potential interaction between microglia (red), myelin (orange), and astrocytes (green). Very fine ramified microglial processes can be seen in the upper left (arrow). Microglia can be observed in close apposition with myelin (^∗^) and astrocytes (^∧^) (Scale bar = 50 μm).

The majority of microglia present in all cultures were in ramified/resting states as shown in Figures 3D–F. The staining intensity for Iba1 in these control cultures on glass is relatively weak, which was expected as the microglia in culture conditions without insult (inflammatory or wound conditions) should not be activated. A notable observation is that the ramified branches of the microglia in the DMMC culture had a fluorescent intensity similar to that seen in the cytoplasm. Conversely in co-culture the ramified branches tended to be of a lower intensity, which suggests there are slight differences in their activation state. Figure 4 shows the interaction between the three glial cells present in the concurrent co-cultures. It reveals a range of potential astrocyte, microglia, and oligodendrocyte interactions that are reflective of those observed *in vivo* (Domingues et al., 2016; Kiray et al., 2016). Note that staining of neural axons was excluded for clarity of the glial cell morphologies.

**FIGURE 5:**
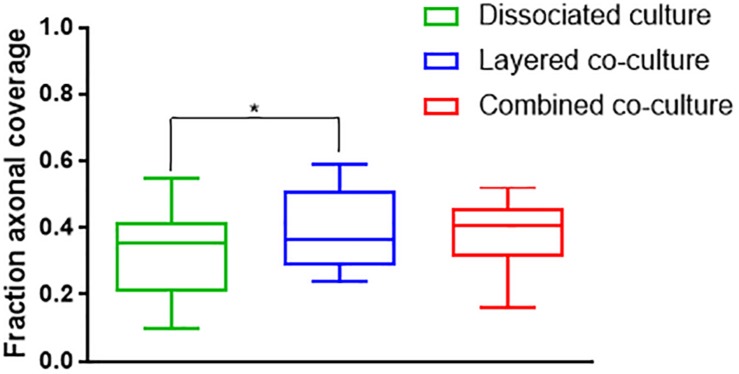
Assessment of average phosphorylated neurofilament coverage as fraction of total image area. Data acquired at 35 days in co-culture (*n* = 3, ^∗^*p* < 0.05).

**FIGURE 6:**
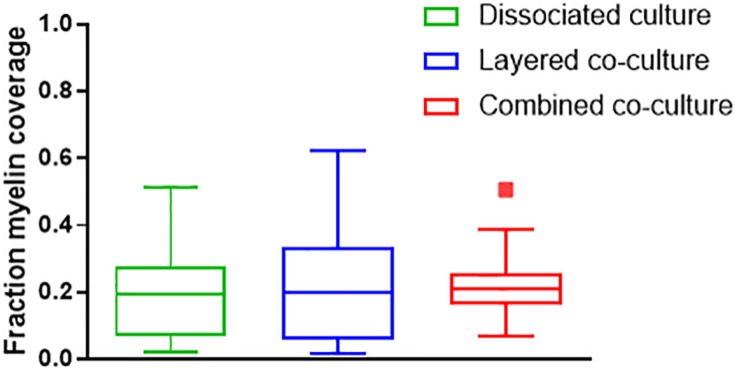
Assessment of average myelin coverage as fraction of total image area inferred from PLP/DM20 positive staining. Data acquired at 35 days in co-culture (*n* = 3).

Representative images of the density and organization of axons (H-NF) and myelin (PLP/DM20) are shown in Figures 3G–L. The addition of mature MGC to the co-cultures did not alter the organization of the H-NF positive axons at 35 days in culture, however both co-cultures had marginally increased axonal coverage, as summarized in Figure 5 compared to the DMMC alone. This was significant for the layered co-culture (*p* < 0.05) when compared with the DMMC culture. However, the contiguity of the staining was more homogenous along the lengths of the axons in the concurrent co-culture. This uniformity of axonal staining in the concurrent co-culture correlated with more consistent myelination along the lengths of the axons as shown in Figure 3L. The myelin coverage in the concurrent co-culture was more consistent when compared with DMMC and layered methods which had greater variance in coverage, as shown in Figure 6.

**FIGURE 7:**
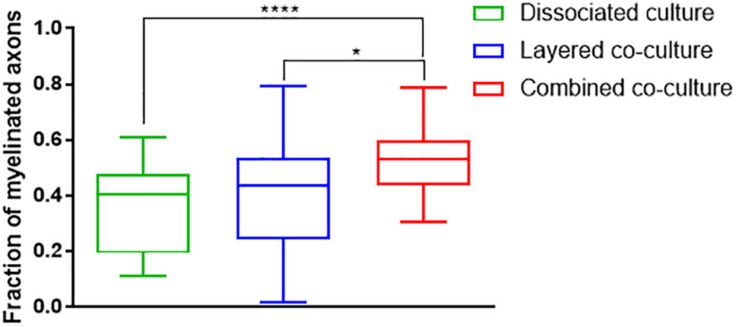
Fraction of Phosphorylated neurofilament which is colocalized with PLP/DM20 used to indicate the proportion of axonal area with a myelin sheath. Data acquired at 35 days in co-culture (*n* = 3, ^∗^*p* < 0.05, ^****^p < 0.0001).

**FIGURE 8:**
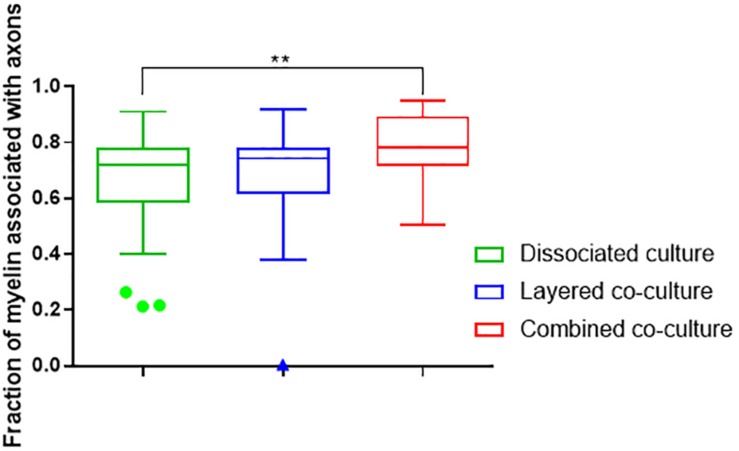
Fraction of PLP/DM20 associated with phosphorylated neurofilament. Data acquired at 35 days in co-culture (*n* = 3, ^∗∗^*p* < 0.01).

The colocalization of H-NF and PLP/DM20 staining yielded two important features which are directly relevant to the level of maturation and health of the cultures. Firstly, the fraction of myelinated axons as described in Figure 7. The concurrent co-culture method consistently generated greater levels of axonal myelination when compared to the DMMC (*p* < 0.0001) and layered co-culture (*p* < 0.05). Secondly, the fraction of myelin produced which is associated with the axons as described in Figure 8, where lower values indicate the oligodendrocytes are less mature and are likely to be in a pre-myelinating state. Consequently, higher values indicate more mature oligodendrocytes, and indirectly more mature axons. The concurrent co-culture had significantly higher levels of myelinated axons compared to the DMMC (*p* < 0.01). The difference in myelination between concurrent and layered methods was not significant, although the layered co-culture exhibited greater variance between replicates.

### Gene Expression – Comparison With *in vivo* CNS Tissue

To enable comparisons between the cultures and the mature *in vivo* mouse CNS, qPCR was performed on mRNA extracted at 21, 28, and 35 days in co-culture and from samples of whole brain. Where possible the qPCR primers were designed for the same targets that were used for immunofluorescence.

**FIGURE 9:**
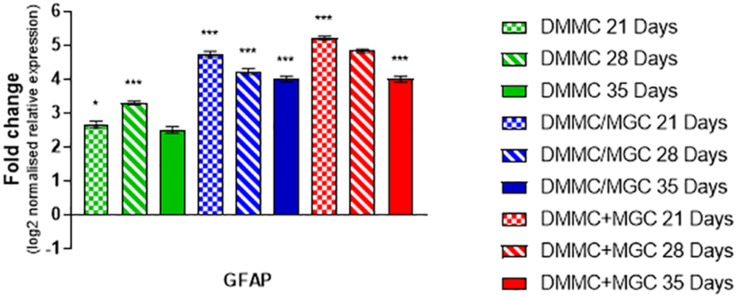
Comparisons of GFAP gene expression relative to healthy adult mouse brain extract. Statistical comparisons relative to reference brain extract shown on graph only (*n* = 3, ^∗^*p* < 0.05, ^∗∗∗^*p* < 0.001).

Figure 9 compares the individual cultures to the *in vivo* mRNA expression of GFAP. Both co-cultures had at least fourfold more GFAP present than the whole brain control at all assessment time points. The DMMC culture had at least 2.5-fold higher expression than the brain extract. This indicates radial glia and/or immature astrocytes were possibly present in both DMMC and co-cultures. Both co-cultures had significantly more GFAP mRNA at 21, 28, and 35 days compared to the DMMC alone, with differences being greater than twofold at 21 days. At subsequent time points the GFAP expression difference between the DMMC and both co-cultures decreased to 1.5-fold (*p* < 0.001). Despite this, the elevated levels of GFAP gene expression did not appear to impact on the levels of H-NF production or myelination. This supports the premise that the increased GFAP expression is in part due to the presence of radial glia or immature astrocytes rather than reactive astrocytes.

**FIGURE 10:**
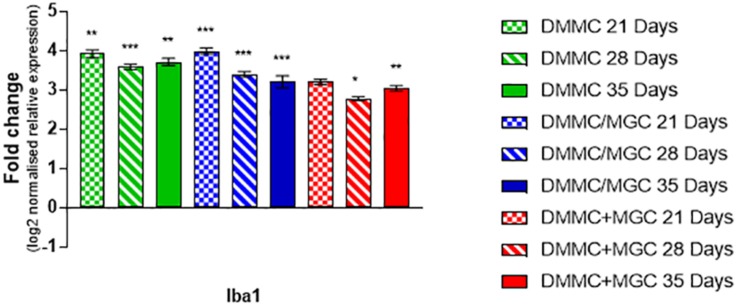
Comparisons of Iba1 gene expression relative to healthy adult mouse brain extract. Statistical comparisons relative to reference brain extract shown on graph only (*n* = 3, ^∗^*p* < 0.05, ^∗∗^*p* < 0.01, ^∗∗∗^*p* < 0.001).

Figure 10 shows that the mRNA expression of Iba1 (microglial inflammatory factor) in the three cultures was at least threefold greater than whole brain extract. However, in contrast to GFAP, the concurrent co-cultures tended to have lower levels of expression when compared to DMMC and layered cultures. The differences between culture types was less than onefold, with the 28 and 35 day concurrent co-cultures being significantly lower than the respective DMMC cultures (*p* < 0.05). The elevated expression is possibly linked to the developmental role of microglia in regulating synapse formation and removal. The discrete differences in expression are in agreement with the small differences in Iba1 staining intensity.

**FIGURE 11:**
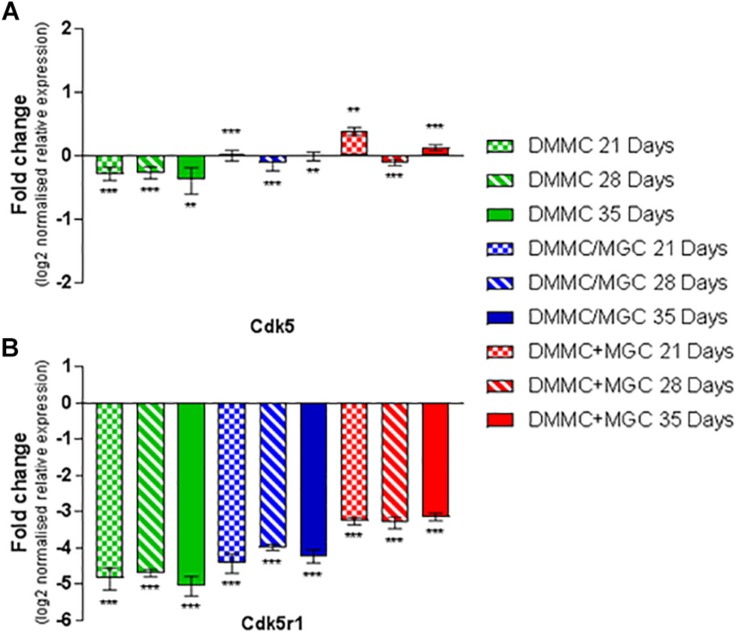
Comparisons of Cdk5 **(A)** and Cdk5r1 **(B)** gene expression relative to healthy adult mouse brain extract. Statistical comparisons relative to reference brain extract shown on graph only (^∗∗^*p* < 0.01, ^∗∗∗^*p* < 0.001).

Cdk5 is expressed in multiple CNS cells including neurons and regulates a diverse range of cellular events. Its expression is required for activation of Cdk5r1 to induce phosphorylation of heavy chain neurofilament expressed in the axonal segment of mature neurons. Figure 11A shows the expression of Cdk5 relative to whole brain mRNA expression. All cultures at all assessment time points are significantly different to the whole brain expression, however the relative differences in are small (<0.4-fold). Conversely, the expression of Cdk5r1 as shown in Figure 11B is at least 4.5-fold less (*p* < 0.001) in DMMC cultures when compared to whole brain extract whereas, layered co-cultures are at least fourfold less (*p* < 0.001) and the concurrent co-cultures are 3 to 3.3-fold less (*p* < 0.001).

The DMMC culture had the lowest expression of both Cdk5 and Cdk5r1 when compared to the co-culture techniques. There were no differences found between Cdk5 expression between the layered and concurrent co-culture methods. For Cdk5r1, the concurrent co-culture expression was at least onefold greater than DMMC cultures (*p* < 0.05 at 21 days and *p* < 0.001 at 28 and 35 days). Most notable though was that the concurrent co-cultures showed consistent expression of Cdk5r1 over all time points, whereas both DMMC and layered cultures showed signs of downregulation at 35 days. This trend suggests the concurrent co-culture produced increased phosphorylated neurofilament formation when compared to the DMMC and layered cultures.

**FIGURE 12:**
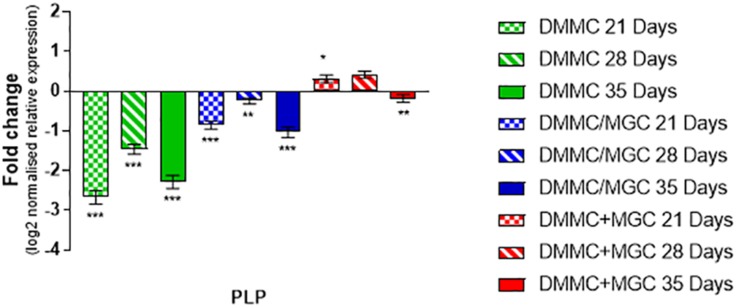
Comparison of PLP gene expression relative to healthy adult mouse brain extract. Statistical comparisons relative to reference brain extract shown on graph only (^∗^*p* < 0.05, ^∗∗^*p* < 0.01, ^∗∗∗^*p* < 0.001).

The production and phosphorylation of heavy chain neurofilament resulting in mature axon formation is indirectly linked to oligodendrocyte maturation and myelination (Jakovcevski et al., 2007; Simons and Nave, 2016). Figure 12 shows the relative expression of PLP mRNA in the three culture types relative to whole brain mRNA expression. Importantly, at all assessment time points the concurrent co-culture had similar levels of PLP expression when compared with the whole brain extract. The concurrent co-culture was shown to have significantly greater PLP expression when compared to both DMMC (*p* < 0.001) and layered (*p* < 0.05) cultures. The mRNA expression of Cdk5r1 and PLP was found to support the morphological data in terms of continuity of H-NF staining in axons and the level of myelination. These results suggest that the concurrent co-culture produced a more consistent culture across the culture period and developed a mature myelinating neural network at an earlier time point. One concern with the PLP expression in the DMMC culture and layered co-culture is there was a measurable downregulation between 28 and 35 days, suggesting possible degeneration.

## Discussion

The two co-culture approaches using frozen MGC cultures were proposed to reduce the total culture time and reliability of the co-culture system for modeling the CNS. Relative to the previous continuous co-culture method which required 45 days to develop mature myelinated neural networks, the concurrent and layered required significantly less time, 25 and 35 days respectively. Compared to the DMMC the concurrent co-culture required two additional steps and four more days to develop. In addition, as MGC were only revived once a mouse was successfully time mated this resulted in 100% success rate for the modified co-culture methods significantly reducing animal breeding costs. Both co-culture approaches resulted in dense networks of myelinated axons with closely associated astrocytes and microglia. The freeze-thaw process on the MGC had no identifiable impact on the subsequent co-cultures. Most notably the shorter recovery time for the MGC in the concurrent co-culture approach was associated with a greater amount of myelinated neural networks at 35 days when compared with the layered and DMMC cultures. Although image analysis of the cultures revealed little difference between the co-cultures and the original DMMC culture with respect to total myelin and H-NF coverage, the concurrent co-culture resulted in increased myelination of axons. Assessment of GFAP did not reveal any notable differences between the culture types. The morphology of the Iba1 stained microglia indicated subtle differences between the DMMC and co-cultures. The microglia in both co-cultures appeared more ramified, thus suggesting a greater level of microglia maturity. This supports the hypothesis that the combination of MGC and DMMC would result in a more mature culture representative of normal CNS tissue *in vivo.*

The relative maturity of astrocytes plays a pivotal role in both neural network development and their ability to undergo reactive astrogliosis (Smith et al., 1990). *In vivo* the differentiation and maturation of astrocytes occurs via reciprocal maturation signals between astrocytes and neurons (Hasel et al., 2017). The time for which astrocytes are cultured prior to interaction with neurons and immature oligodendrocytes, impacts on their ability to myelinate axons (Ishikawa et al., 1996). Additionally, astrocyte maturity has been shown to directly impact oligodendrocyte differentiation (Ishikawa et al., 1996; Nash, 2010; Nash et al., 2011a), with increased time in isolated culture resulting in inhibition of myelination, as a consequence of absent cues from the developing neurons. Although there are no apparent differences in the GFAP morphologies present between the culture types, there are significant differences at the mRNA level. At 21 days both co-cultures had greater than twofold more GFAP mRNA relative to the DMMC culture alone. This difference decreased to 1.5-fold at 35 days. Elevated GFAP is classically associated with reactive gliosis associated with neurotrauma, diseases, or neurodegeneration (Ridet et al., 1997; Silver and Miller, 2004; Middeldorp and Hol, 2011; Gao et al., 2013; Brenner, 2014; Burda and Sofroniew, 2014; Cregg et al., 2014; Pekny et al., 2014; Liddelow and Barres, 2017). However, despite the increased mRNA expression of GFAP and its changes over time in culture, the increased levels had no measurable impact on the processes of axonal growth and phosphorylation of H-NF and subsequent myelination in the co-cultures.

In light of the apparent lack of impact of the elevated GFAP on neural network development, indicates there are a number of possible explanations for the elevated GFAP expression compared to the *in vivo* tissues. Firstly, the site of mRNA extraction from the CNS carries potential variability. *In vivo* there is regional heterogeneity in GFAP positive astrocytes (Schitine et al., 2015) which results in differential expression levels of GFAP. The *in vivo* tissue collection site relative to the *in vitro* cell population could be inherently different. Secondly, the astrocytes in the culture are likely in a mild inflammatory state resulting in increased GFAP expression (Liddelow and Barres, 2017), this is a consequence of being grown on rigid substrates such as glass and tissue culture plastic (Wilson et al., 2016). Alternatively, it is possible that this difference is an additive result of the two component cultures, DMMC and MGC, contributing to the mRNA expression, which is partly supported by the relative increase in GFAP expression of the co-cultures over the DMMC alone. Further to this, at 7 days in culture the DMMC likely consists of GFAP positive radial glia which continue to divide and differentiate into mature astrocytes (McDermott et al., 2005) and non-astrocytic cells. *In vivo* radial glial become prevalent in the mouse spinal cord tissue around E9.5 days (Hall and Miller, 2012) and undergo differentiation into immature astrocytes between E18 and P14 days of age (in rodents) (Reemst et al., 2016). This timeline correlates to a peak differentiation of the radial glia into astrocytes and other cell types around 10–14 days in culture from the DMMC population. Further to this, *in vivo* data from Riol et al. (1992) described initial increases in GFAP mRNA levels from P0 to P20 days followed by declining levels out to P60 days. Both co-cultures appeared to follow this trend after 21 days and the DMMC after 28 days. This suggests that the co-cultures develop at a faster rate compared to the DMMC. However further research is required to map this change over the entire culture period to determine the exact difference in development time between the culture types, and how this relates to *in vivo* CNS development.

In conjunction with the elevated mRNA levels of GFAP, Iba1 was also at least threefold higher compared to whole brain mRNA in all culture types. Although only minor differences in expression were found between the culture types, the concurrent co-culture exhibited the lowest level of relative Iba1 expression. This elevated mRNA expression compared to whole brain was contrasted by the dominant ramified morphologies present in the co-cultures, which indicates a healthy, mature resting state (Lively and Schlichter, 2013; Ferreira and Bernardino, 2015). This increased Iba1 mRNA expression in the cultures relative to the adult mouse brain is potentially associated with the developmental roles of microglia in regulating synapse formation via pruning of unnecessary connections (Chaboub and Deneen, 2013; Tay et al., 2017). However, continuous co-cultures, described in Gilmour (2018) which were grown on different materials indicated the microglia are capable of maintaining resting phenotypes on control materials or taking on activated phenotypes in response to test materials, thus suggesting the elevated mRNA levels might not be due to immature microglia. It is also possible that the elevated mRNA is an artifact of the 2D culture format, combined with the physiological irrelevant volume of media required to maintain the metabolic requirements of the cultures. The effect of media volume and culture format has been shown previously to have significant effects on osteocytes (Yoshimura et al., 2017) and hepatocytes (Haque et al., 2016).

The phosphorylation of neurofilament is controlled through Cdk5 and the neuron specific activator Cdk5r1 (Wang et al., 2012). At 21 days in culture, the concurrent co-culture expressed significantly more Cdk5 compared to the other cultures, but this difference decreased at 28 and 35 days. As Cdk5 is associated with other processes and cell types within the developing and mature CNS (Zhu et al., 2011) the expression of Cdk5r1 combined with Cdk5 is more relevant. *In vitro* Cdk5r1 expression was significantly higher in the concurrent co-cultures at all time points, except 21 days when compared to the layered co-culture. This increased co-expression of Cdk5/Cdk5r1 did not result in a greater number of axons, but the axonal expression of the phosphorylated neurofilament was more contiguous. This infers the axonal processes in concurrent co-cultures are more stable and more resistant to degeneration (Sun et al., 1996; Zhu et al., 2011). The increased stability of the neural processes could be indirectly linked to the lower levels of Iba1 in the concurrent co-cultures as there is less phagocytosis of degraded axons (Ekdahl, 2012).

Comparing the production of phosphorylated neurofilament in the co-cultures revealed Cdk5r1 expression was at least threefold less in concurrent co-cultures and fourfold less in both DMMC and layered cultures compared to whole brain extract. The difference between *in vivo* and *in vitro* expression could be the result of the 2D nature of the culture environment (Zare-Mehrjardi et al., 2011). The 2D environment limits the total number of axons and axon length, an observation similar to that previously made by Sun et al. (2016) in reference to the differences between 2D and 3D neural cell cultures. Although there is less Cdk5r1 *in vitro* than *in vivo*, there are similar levels of Cdk5. This is likely due to a secondary role of Cdk5 in modulating OPC differentiation into oligodendrocytes (Miyamoto et al., 2007). The concurrent co-cultures expressed similar amounts of PLP mRNA compared to adult brain extract, which correlates with the expression of Cdk5 for all culture types and time points. It has been proposed that Cdk5 interacts with OPCs promoting differentiation, although via different pathways to neurofilament phosphorylation (Miyamoto et al., 2007), but is facilitated as a secondary effect of this interaction (Yang et al., 2013b; Luo et al., 2016). Taken together the relative expression of Cdk5 and PLP is likely linked to the differentiation of OPCs into mature oligodendrocytes (Miyamoto et al., 2007), as all culture methods resulted in similar amounts of total myelin. However, the concurrent co-culture resulted in a higher level of myelin associated with axons and subsequently more myelinated axons. The process of axon myelination is complex an only partially understood, but is thought to be governed first by intrinsic actions followed by adaptive changes (Bechler et al., 2018). Oligodendrocytes have been shown to intrinsically wrap axons and axon like structures (Rosenberg et al., 2008; Tuck et al., 2016), however this initial myelination is transient unless stabilized through adaptive changes. The adaptive stabilization process is hypothesized to only occur based on interactive signals from active mature axons (Almeida, 2018). This might indicate that the combined co-culture has more mature neurons resulting in stabilized myelin sheaths compared with the layered approach.

Contrary to the expected relationship between GFAP, Cdk5/Cdk5r1 and PLP expression, the concurrent co-culture expressed the highest levels of GFAP at 21 and 28 days in culture. These time points were also associated with the highest level of axonal myelination. It was anticipated that the higher levels of GFAP expression would be associated with lower production of H-NF and PLP. *In vivo* H-NF is primarily found in its phosphorylated form in mature axons within the adult CNS (Wang et al., 2012) and is sparse in the developing and immature CNS (Haque et al., 2016). At present there is no known explanation for this relationship.

Although the model presented here does not include the blood–brain barrier (BBB) or peripheral immune cells, which are critical components of the *in vivo* response to intracortical implants and traumatic CNS injury (Polikov et al., 2005; Groothuis et al., 2014). The objective of this work was to establish a robust, rapidly maturing co-culture of the CNS which has the potential to replicate some of the hallmarks of CNS injury. Although BBB disruption is one of the key attributes of traumatic CNS injury, recent literature indicates the interplay of the peripheral immune system has greater impacts in wound progression and secondary degeneration (Evans et al., 2014; Ertürk et al., 2016; Makinde et al., 2017; Abe et al., 2018). Future studies could expand on this model through the inclusion of peripheral immune cells or immune cell conditioned media (Haan et al., 2015) at different developmental or post-insult time points to evaluate the mechanisms of how the peripheral immune system alters CNS behavior in development and after insult.

Within the limitations of a 2D model to represent the 3D *in vivo* CNS, co-culturing mature MGC and DMMC populations provides a promising platform for modeling multicellular behaviors and responses to exogenous stimuli. The inclusion of a more mature glial cell population enables the culture to react in a more *in vivo* mimetic way. This was demonstrated in our previous research, whereby the inclusion of mature astrocytes dramatically altered the response of neural cell development and oligodendrocyte differentiation in response to different materials. The concurrent co-culture method provides a good robust model for use in wound healing studies and biomaterial assessment often conducted on less relevant culture systems. The combined co-culture improves on existing models by enabling the formation of mature neural networks within 25 days, compared to alternative methods which take > 5 weeks to reach maturity. In addition, the model does not require exogenous ECM coating of growth surfaces for cell attachment, as ECM type can affect neural progenitor differentiation and cell migration, thus impacting the overall cell behavior (Ma et al., 2008). The co-culture is completely serum free after 12 days in culture. The serum free nature enables evaluation of the cultures at the proteomic level without the confound of animal sera. Lastly approximately 150 cultures can be obtained from 2 neonatal and 6 E13.5 embryonic mice in a 24 well format.

Future work will determine to what extent the combined co-culture model is able to replicate cell behaviors relevant and consistent with the *in vivo* CNS injury. To achieve this, it is necessary to analyze the expression of pro and anti-inflammatory cytokines and chemokines present within the cultures, relative to the native CNS in conjunction with genetic and morphological analysis.

## Conclusion

The modified co-cultures both substantially increased the reliability and repeatability of the co-culture method. When the co-cultures were compared at the genotypic and phenotypic levels to the DMMC culture method, both methods resulted in improved and more rapid myelinated neural network development. The concurrent co-culture where MGCs were plated at the same time as DMMCs, performed the most consistently over all experimental repeats with reference to axonal coverage and myelination. Although both co-cultures had elevated GFAP and Iba1 mRNA expression at all time points relative to the DMMC this did not impact on the neural network development.

Comparing the co-cultures to whole brain extract, the layered co-culture expressed significantly decreased levels of myelin and Cdk5r1 resulting in lower neurofilament phosphorylation. The concurrent co-culture on the other had had significantly increased production of myelin similar to *in vivo* levels. Although it had lower levels of neurofilament phosphorylation relative to the whole brain control, although this was expected due to the spatial and ECM limitations of a 2D model. The concurrent co-culture showed consistent levels of PLP, Cdk5 and Cdk5r1 indicating that 21 days was sufficient to be considered a mature 2D *in vitro* model of the CNS. The concurrent co-culture method may provide a viable *in vitro* pre-clinical tool for assessing CNS cell responses as it mimics a more comprehensive number of properties of the mature healthy CNS, than existing *in vitro* models. Future work will characterize the concurrent co-culture response to physical injury and control biomaterials in order to assess its use as a tool for high-throughput pre-clinical testing of neural interfacing biomaterials.

## Data Availability Statement

The raw data supporting the conclusions of this article will be made available by the authors, without undue reservation, to any qualified researcher.

## Ethics Statement

The animal study was reviewed and approved by the University of New South Wales Animal Care and Ethics Committee.

## Author Contributions

The research studies presented herein were the work of AG during his doctoral studies, supervised by LP-W and RG. The manuscript preparation was undertaken by AG in consultation and with direct contribution from LP-W and RG.

## Conflict of Interest

The authors declare that the research was conducted in the absence of any commercial or financial relationships that could be construed as a potential conflict of interest.
